# State-Specific Prevalence of Inactivity, Self-Rated Health Status, and Severe Joint Pain Among Adults With Arthritis — United States, 2019

**DOI:** 10.5888/pcd19.210346

**Published:** 2022-04-28

**Authors:** Lindsey M. Duca, Charles G. Helmick, Kamil E. Barbour, Louise B. Murphy, Dana Guglielmo, Erica L. Odom, Michael A. Boring, Janet B. Croft

**Affiliations:** 1 Epidemic Intelligence Service, Centers for Disease Control and Prevention, Atlanta, Georgia; 2 Division of Population Health, National Center for Chronic Disease Prevention and Health Promotion, Centers for Disease Control and Prevention, Atlanta, Georgia; 3 Optum Life Sciences, Inc, Eden Prairie, Minnesota; 4 Oak Ridge Institute for Science and Education, Oak Ridge, Tennessee

## Abstract

Arthritis is associated with joint pain, disability, and physical inactivity, potentially resulting in poor quality of life. The Centers for Disease Control and Prevention analyzed 2019 Behavioral Risk Factor Surveillance System data to estimate state-specific arthritis prevalence and, among adults with arthritis, the prevalence of physical inactivity, fair/poor self-rated health status, and severe joint pain. Among adults with arthritis, age-standardized prevalences of physical inactivity, fair/poor health status, and severe joint pain were high in all states and highest in southeastern states. Increased promotion and use of evidence-based public health interventions for arthritis may improve health-promoting behaviors and health outcomes among adults with arthritis.

**Summary**

**What is already known on this topic?**

Arthritis is associated with joint pain and physical inactivity, potentially resulting in poor quality of life. Little is known about geographic variations in the prevalence of physical inactivity, fair/poor health status, and severe joint pain among adults with arthritis.

**What is added by this report?**

In 2019 the prevalence of arthritis was highest in Appalachia. Among adults with arthritis, prevalence of physical inactivity, fair/poor health status, and severe joint pain were high in all states and highest in southeastern states.

**What are the implications for public health practice?**

High burden of physical inactivity, fair/poor health status, and severe joint pain warrant greater awareness and increased use of arthritis-appropriate, evidence-based public health interventions.

## Objective

Arthritis is a common condition, a common comorbidity, and a leading cause of disability in the US (1). For arthritis, benefits of nonpharmacologic interventions, such as physical activity and self-management education, include improved self-rated health status, physical functioning, physical activity levels, confidence in one’s ability to manage chronic conditions, and mood; benefits can also include reduced joint pain, fatigue, or functional limitations (2–4). We examined age-standardized state-specific arthritis prevalence and, among adults with arthritis, the prevalence of 3 arthritis-related characteristics — physical inactivity, fair/poor self-rated health status, and severe joint pain.

## Methods

We analyzed 2019 Behavioral Risk Factor Surveillance System (BRFSS) data to estimate state-specific prevalence of physical inactivity, fair/poor health status, and severe joint pain among adults with arthritis. In 2019, BRFSS, an annual state-based telephone survey, collected data among the noninstitutionalized US population aged 18 years or older from 49 states (New Jersey did not collect sufficient data), the District of Columbia, and the US territories of Guam and Puerto Rico. Estimates were age-standardized to the 2000 US projected population aged 18 years or older by using 3 age groups: 18 to 44 years, 45 to 64 years, and 65 years or older (https://www.cdc.gov/nchs/data/statnt/statnt20.pdf). Arthritis was defined as a response of yes to “Have you ever been told by a doctor or other health care professional that you have arthritis, rheumatoid arthritis, gout, lupus, or fibromyalgia?” Among adults with arthritis, physical inactivity was defined as a no response to “During the past month, other than your regular job, did you participate in any physical activities or exercises such as running, calisthenics, golf, gardening, or walking for exercise?” Fair/poor self-rated health status was defined as a “fair” or “poor” response to “Would you say that in general your health is excellent, very good, good, fair, or poor?” Severe joint pain was considered a rating of 7 to 10 (on a 0 to 10 scale) when asked how bad their joint pain was on average during the past 30 days.

State-specific weighted unadjusted and age-standardized (5) prevalence estimates with 95% CIs were estimated for arthritis and, among adults with arthritis, for the 3 arthritis-related characteristics (physical inactivity, fair/poor self-rated health status, and severe joint pain). In 2019, the landline and cellular telephone (combined) median state survey response rate was 49.4% (range, 37.3% to 73.1%) (6). All analyses accounted for the complex sampling design of the survey, and sampling weights were generated by using iterative proportional fitting and applied to make state-specific estimates representative of each state. Analyses were conducted by using SAS (version 9.4; SAS Institute) and SUDAAN (version 11.0; RTI International).

## Results

In 2019, for the 49 states and the District of Columbia, the unadjusted median prevalence of arthritis was 26.1% (range: 17.2% in the District of Columbia to 41.4% in West Virginia) (Table). Across states, the median age-standardized prevalence of arthritis was 23.6% (range: 18.4% in California and Hawaii to 36.4% in West Virginia) with the Appalachia region (ie, residents of part or all of Alabama, Georgia, Kentucky, Maryland, Mississippi, New York, North Carolina, Ohio, Pennsylvania, South Carolina, Tennessee, Virginia, and West Virginia) having the highest prevalence (Figure).

**Table:** Unadjusted, State-Specific Prevalence of Arthritis^a^ Among Adults Aged ≥18 Years and, Prevalence Among Those Adults With Arthritis With Physical Inactivity^b^, Fair/Poor Self-Rated Health Status^c^, and Severe Joint Pain^d^, in 49 States^e^, the District of Columbia, Guam, and Puerto Rico — Behavioral Risk Factor Surveillance System, 2019^f^

State or area	Prevalence of arthritis		Adults with arthritis		
	Weighted number^g^	Unadjusted % (95% CI)	% Reporting physical inactivity, unadjusted (95% CI)	% Reporting fair/poor self-rated health status, unadjusted (95% CI)	% Reporting severe joint pain, unadjusted (95% CI)
Alabama	1,273,000	33.9 (32.5–35.3)	39.9 (37.6–42.3)	40.6 (38.3–42.9)	41.3 (39.0–43.6)
Alaska	116,000	21.4 (19.4–23.5)	27.0 (22.2–32.4)	31.3 (27.0–36.1)	25.1 (20.7–30.0)
Arizona	1,301,000	23.6 (22.2–24.9)	32.6 (29.7–35.7)	36.2 (33.3–39.2)	32.2 (29.3–35.2)
Arkansas	715,000	31.2 (29.6–32.9)	41.5 (38.6–44.5)	44.5 (41.6–47.5)	41.2 (38.3–44.2)
California	6,007,000	19.8 (18.9–20.7)	26.3 (24.1–28.6)	31.9 (29.6–34.3)	30.2 (27.9–32.6)
Colorado	990,000	22.3 (21.4–23.2)	22.4 (20.5–24.4)	26.9 (24.9–29.0)	25.7 (23.7–27.8)
Connecticut	653,000	23.5 (22.5–24.6)	29.9 (27.7–32.3)	27.9 (25.7–30.2)	26.4 (24.3–28.7)
Delaware	208,000	27.4 (25.6–29.3)	36.7 (33.0–40.5)	29.4 (25.9–33.1)	31.7 (28.0–35.7)
District of Columbia	97,000	17.2 (15.7–18.9)	32.8 (28.1–37.9)	34.1 (29.7–38.7)	36.5 (31.8–41.4)
Florida	4,325,000	25.4 (24.1–26.7)	32.8 (30.2–35.5)	35.2 (32.5–37.9)	36.5 (33.8–39.2)
Georgia	1,902,000	23.8 (22.4–25.2)	36.2 (33.2–39.2)	37.1 (34.1–40.2)	35.2 (32.3–38.1)
Hawaii	230,000	20.9 (19.8–22.1)	27.4 (24.7–30.2)	28.0 (25.4–30.7)	23.4 (20.9–26.0)
Idaho	329,000	25.1 (23.4–26.8)	29.6 (26.2–33.1)	35.2 (31.6–38.9)	30.1 (26.7–33.9)
Illinois	2,409,000	24.7 (23.5–26.0)	31.6 (28.9–34.3)	32.2 (29.5–35.0)	30.0 (27.3–32.7)
Indiana	1,358,000	26.9 (25.9–28.0)	39.8 (37.5–42.1)	39.7 (37.5–41.9)	32.8 (30.7–35.0)
Iowa	618,000	25.7 (24.7–26.6)	31.7 (29.8–33.7)	27.5 (25.6–29.4)	23.8 (22.1–25.7)
Kansas	555,000	25.6 (24.7–26.5)	33.7 (31.7–35.7)	33.9 (31.9–35.9)	30.2 (28.2–32.2)
Kentucky	1,176,000	34.3 (32.7–35.9)	42.8 (40.0–45.7)	40.2 (37.5–43.0)	37.2 (34.5–40.0)
Louisiana	968,000	27.6 (26.1–29.2)	39.0 (35.9–42.1)	42.1 (39.0–45.2)	43.5 (40.4–46.7)
Maine	340,000	31.8 (30.5–33.1)	37.1 (34.8–39.4)	32.9 (30.6–35.2)	27.6 (25.5–29.8)
Maryland	1,107,000	23.9 (23.1–24.8)	29.4 (27.7–31.1)	26.5 (24.9–28.2)	30.1 (28.3–31.9)
Massachusetts	1,316,000	24.5 (23.3–25.7)	31.7 (29.2–34.3)	28.6 (26.2–31.2)	26.8 (24.4–29.4)
Michigan	2,373,000	30.8 (29.6–31.9)	33.1 (31.1–35.2)	32.3 (30.2–34.3)	31.3 (29.2–33.3)
Minnesota	928,000	21.7 (20.9–22.4)	24.8 (23.2–26.5)	26.7 (25.0–28.4)	23.2 (21.6–24.9)
Mississippi	650,000	28.8 (27.3–30.4)	47.5 (44.5–50.4)	44.5 (41.6–47.4)	44.0 (41.1–47.0)
Missouri	1,270,000	27.1 (25.8–28.4)	36.6 (34.1–39.2)	36.3 (33.8–38.9)	33.4 (30.9–36.0)
Montana	241,000	28.9 (27.7–30.2)	25.8 (23.6–28.0)	29.9 (27.6–32.3)	23.4 (21.3–25.7)
Nebraska	335,000	23.1 (22.3–24.0)	33.4 (31.5–35.3)	28.5 (26.7–30.3)	23.5 (21.8–25.3)
Nevada	531,000	22.7 (20.6–25.0)	29.4 (24.6–34.7)	36.2 (30.9–41.8)	32.2 (27.4–37.6)
New Hampshire	287,000	26.4 (25.0–27.9)	28.1 (25.4–31.0)	27.7 (25.1–30.4)	27.0 (24.3–29.8)
New Mexico	413,000	25.8 (24.4–27.3)	30.9 (28.0–33.9)	35.5 (32.6–38.6)	35.4 (32.4–38.5)
New York	3,302,000	22.1 (21.2–23.0)	31.9 (29.8–34.1)	32.4 (30.3–34.5)	32.6 (30.5–34.8)
North Carolina	2,172,000	27.0 (25.5–28.5)	33.2 (30.1–36.5)	35.2 (32.1–38.4)	36.3 (33.2–39.6)
North Dakota	147,000	25.4 (23.9–26.9)	32.1 (29.2–35.0)	27.0 (24.3–29.9)	21.1 (18.6–23.8)
Ohio	2,751,000	30.6 (29.5–31.8)	37.1 (34.9–39.3)	36.3 (34.2–38.5)	32.1 (30.1–34.2)
Oklahoma	790,000	27.0 (25.7–28.3)	39.5 (37.0–42.1)	40.1 (37.5–42.7)	35.5 (33.0–38.2)
Oregon	863,000	26.3 (25.0–27.6)	26.8 (24.3–29.5)	31.9 (29.1–34.7)	25.0 (22.5–27.6)
Pennsylvania	2,910,000	29.1 (27.7–30.5)	32.5 (30.0–35.2)	32.5 (30.0–35.0)	30.5 (28.0–33.1)
Rhode Island	224,000	26.8 (25.3–28.3)	31.8 (29.0–34.6)	29.8 (27.2–32.6)	32.3 (29.5–35.2)
South Carolina	1,114,000	28.2 (26.9–29.5)	36.4 (33.8–39.1)	38.1 (35.5–40.7)	37.3 (34.7–39.9)
South Dakota	176,000	26.7 (24.6–28.9)	39.3 (34.9–43.9)	32.8 (28.4–37.5)	23.1 (19.5–27.3)
Tennessee	1,598,000	30.6 (29.1–32.2)	40.1 (37.3–42.9)	42.1 (39.3–44.8)	38.5 (35.8–41.3)
Texas	4,398,000	20.7 (19.5–22.0)	33.5 (30.4–36.6)	37.6 (34.5–40.7)	36.0 (32.9–39.1)
Utah	519,000	23.1 (22.2–24.0)	22.2 (20.5–24.0)	26.1 (24.3–28.0)	22.4 (20.6–24.2)
Vermont	135,000	27.0 (25.6–28.6)	29.3 (26.6–32.1)	26.5 (23.9–29.3)	25.5 (22.9–28.2)
Virginia	1,730,000	26.3 (25.2–27.4)	33.0 (30.8–35.2)	32.3 (30.1–34.5)	30.1 (28.0–32.4)
Washington	1,439,000	24.6 (23.7–25.5)	23.3 (21.5–25.1)	29.6 (27.7–31.5)	23.4 (21.7–25.2)
West Virginia	585,000	41.4 (39.7–43.1)	39.1 (36.8–41.5)	43.2 (40.8–45.7)	37.3 (35.0–39.8)
Wisconsin	1,244,000	27.8 (26.3–29.3)	31.0 (28.2–34.1)	29.2 (26.4–32.2)	25.4 (22.6–28.4)
Wyoming	109,000	25.1 (23.5–26.8)	30.6 (27.4–34.0)	33.3 (30.0–36.8)	26.3 (23.2–29.7)
49 states and District of Columbia median (range)	Not applicable	26.1 (17.2–41.4)	32.6 (22.2–47.5)	32.6 (26.1–44.5)	30.4 (21.1–44.0)
Guam	17,000	16.1 (14.0–18.5)	39.9 (32.6–47.7)	43.7 (36.2–51.5)	35.9 (29.1–43.4)
Puerto Rico	574,000	21.2 (20.0–22.4)	59.7 (56.6–62.8)	67.7 (64.7–70.5)	58.2 (55.1–61.3)

a Respondents were classified as having arthritis if they responded yes to the question “Have you ever been told by a doctor or other health care professional that you have arthritis, rheumatoid arthritis, gout, lupus, or fibromyalgia?”

b Physical inactivity was defined by asking the question, “During the past month, other than your regular job, did you participate in any physical activities or exercises such as running, calisthenics, golf, gardening, or walking for exercise?” to which respondents had answered no.

c Respondents were categorized as having fair/poor self-rated health status when answering “fair” or “poor” to the question, “Would you say that in general your health is excellent, very good, good, fair, or poor?”

d Respondents were classified as having severe joint pain if they responded with a rating of 7 to 10 to the question, “Please think about the past 30 days, keeping in mind all of your joint pain or aching and whether or not you have taken medication. During the past 30 days, how bad was your joint pain on average on a scale of 0 to 10 where 0 is no pain and 10 is pain or aching as bad as it can be?”

e In 2019, New Jersey did not collect sufficient data to meet the minimum requirement for inclusion in the Behavioral Risk Factor Surveillance System public-use data set.

f Data presented are prevalence (95% CI) unless otherwise specified.

g Weighted number is the estimated number of adults with arthritis who reported the outcome.

**Figure:**
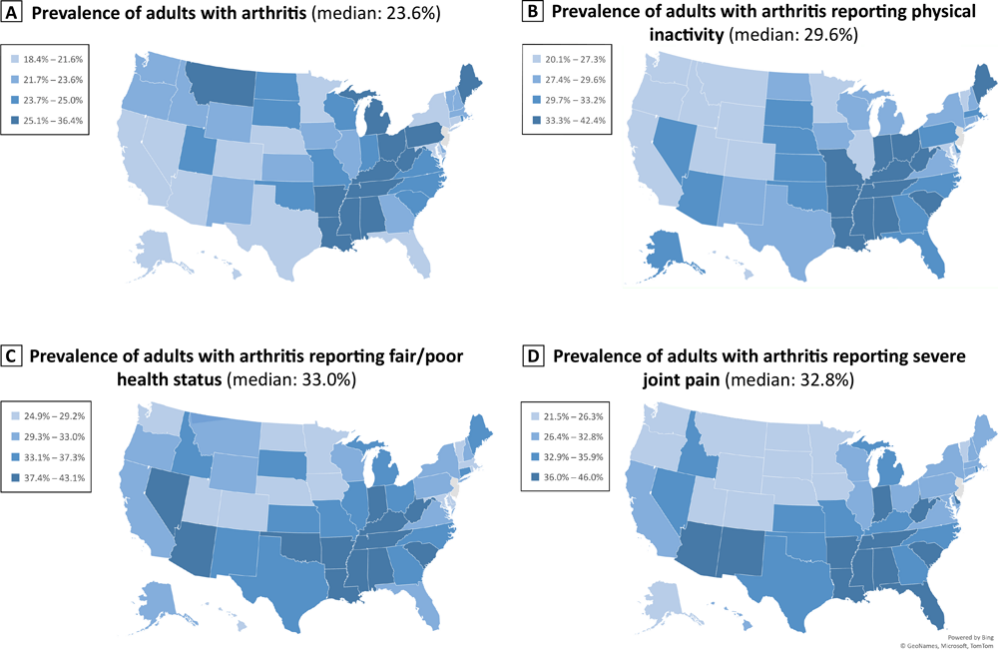
Age-standardized, state-specific prevalence of arthritis among adults aged ≥18 years, and prevalence among those adults with arthritis with physical inactivity, fair/poor self-rated health status, or severe joint pain, in 49 states by quartile — Behavioral Risk Factor Surveillance System, 2019. In 2019, New Jersey did not collect sufficient data to meet the minimum requirement for inclusion in the BRFSS public-use data set. Estimates were age-standardized to the 2000 US projected population aged ≥18 years by using 3 age groups: 18−44 years, 45–64 years, and ≥65 years (https://www.cdc.gov/nchs/data/statnt/statnt20.pdf). Respondents were classified as having arthritis if they responded yes to the question, “Have you ever been told by a doctor or other health care professional that you have arthritis, rheumatoid arthritis, gout, lupus, or fibromyalgia?” Physical inactivity was defined by using the question, “During the past month, other than your regular job, did you participate in any physical activities or exercises such as running, calisthenics, golf, gardening, or walking for exercise?” and respondents answered no. Respondents were categorized as having fair/poor self-rated health status when answering “fair” or “poor” to the question, “Would you say that in general your health is excellent, very good, good, fair, or poor?” Respondents were classified as having severe joint pain if they responded with a rating of 7 to 10 to the question, “Please think about the past 30 days, keeping in mind all of your joint pain or aching and whether or not you have taken medication. During the past 30 days, how bad was your joint pain on average on a scale of 0 to 10 where 0 is no pain and 10 is pain or aching as bad as it can be?”

**Table:** 

State	Arthritis (median, 23.6%)	Physical inactivity (median, 29.6%)	Fair/poor health status (median, 33.0%)	Severe joint pain (median, 32.8%)
Alabama	25.1–36.4	33.3–42.4	37.4–43.1	36.0–46.0
Alaska	18.4–21.6	29.7–33.2	29.3–33.0	21.5–26.3
Arizona	18.4–21.6	29.7–33.2	37.4–43.1	36.0–46.0
Arkansas	25.1–36.4	33.3–42.4	37.4–43.1	36.0–46.0
California	18.4–21.6	20.1–27.3	29.3–33.0	26.4–32.8
Colorado	18.4–21.6	20.1–27.3	24.9–29.2	21.5–26.3
Connecticut	18.4–21.6	27.4–29.6	33.1–37.3	26.4–32.8
Delaware	21.7–23.6	29.7–33.2	24.9–29.2	36.0–46.0
Florida	18.4–21.6	29.7–33.2	29.3–33.0	36.0–46.0
Georgia	21.7–23.6	29.7–33.2	33.1–37.3	32.9–35.9
Hawaii	18.4–21.6	20.1–27.3	24.9–29.2	26.4–32.8
Idaho	21.7–23.6	20.1–27.3	33.1–37.3	32.9–35.9
Illinois	21.7–23.6	20.1–27.3	33.1–37.3	26.4–32.8
Indiana	23.7–25.0	33.3–42.4	37.4–43.1	36.0–46.0
Iowa	21.7–23.6	27.4–29.6	24.9–29.2	21.5–26.3
Kansas	21.7–23.6	29.7–33.2	33.1–37.3	32.9–35.9
Kentucky	25.1–36.4	33.3–42.4	37.4–43.1	32.9–35.9
Louisiana	25.1–36.4	33.3–42.4	37.4–43.1	36.0–46.0
Maine	25.1–36.4	33.3–42.4	33.1–37.3	26.4–32.8
Maryland	18.4–21.6	20.1–27.3	24.9–29.2	26.4–32.8
Massachusetts	21.7–23.6	29.7–33.2	24.9–29.2	26.4–32.8
Michigan	25.1–36.4	27.4–29.6	33.1–37.3	32.9–35.9
Minnesota	18.4–21.6	20.1–27.3	24.9–29.2	21.5–26.3
Mississippi	25.1–36.4	33.3–42.4	37.4–43.1	36.0–46.0
Missouri	23.7–25.0	33.3–42.4	33.1–37.3	32.9–35.9
Montana	25.1–36.4	20.1–27.3	29.3–33.0	21.5–26.3
Nebraska	18.4–21.6	29.7–33.2	24.9–29.2	21.5–26.3
Nevada	18.4–21.6	29.7–33.2	37.4–43.1	32.9–35.9
New Hampshire	21.7–23.6	27.4–29.6	29.3–33.0	26.4–32.8
New Mexico	21.7–23.6	27.4–29.6	33.1–37.3	36.0–46.0
New York	18.4–21.6	27.4–29.6	29.3–33.0	26.4–32.8
North Carolina	23.7–25.0	29.7–33.2	33.1–37.3	32.9–35.9
North Dakota	23.7–25.0	27.4–29.6	24.9–29.2	21.5–26.3
Ohio	25.1–36.4	33.3–42.4	33.1–37.3	26.4–32.8
Oklahoma	23.7–25.0	29.7–33.2	37.4–43.1	32.9–35.9
Oregon	21.7–23.6	20.1–27.3	29.3–33.0	21.5–26.3
Pennsylvania	25.1–36.4	29.7–33.2	29.3–33.0	26.4–32.8
Rhode Island	23.7–25.0	27.4–29.6	24.9–29.2	32.9–35.9
South Carolina	23.7–25.0	33.3–42.4	37.4–43.1	36.0–46.0
South Dakota	23.7–25.0	29.7–33.2	33.1–37.3	21.5–26.3
Tennessee	25.1–36.4	33.3–42.4	37.4–43.1	36.0–46.0
Texas	18.4–21.6	27.4–29.6	33.1–37.3	32.9–35.9
Utah	23.7–25.0	20.1–27.3	24.9–29.2	21.5–26.3
Vermont	21.7–23.6	20.1–27.3	24.9–29.2	21.5–26.3
Virginia	23.7–25.0	27.4–29.6	29.3–33.0	26.4–32.8
Washington	21.7–23.6	20.1–27.3	24.9–29.2	21.5–26.3
West Virginia	25.1–36.4	33.3–42.4	37.4–43.1	36.0–46.0
Wisconsin	23.7–25.0	27.4–29.6	29.3–33.0	26.4–32.8
Wyoming	21.7–23.6	20.1–27.3	29.3–33.0	21.5–26.3

Among adults with arthritis, the median age-standardized prevalence of physical inactivity in the past 30 days was 29.6% (range: 20.1% in Utah to 42.4% in Mississippi) with the southeastern states having the highest prevalence estimates (Figure). The median age-standardized prevalence of fair/poor self-rated health status was 33.0% (range: 24.9% in Hawaii to 43.1% in Arkansas), and prevalences were similarly highest in the southeastern states. The median age-standardized prevalence of severe joint pain was 32.8% (range: 21.5% in North Dakota to 46.0% in Louisiana) with the southeastern states having the highest prevalences.

## Discussion

In 2019 the median unadjusted prevalence of arthritis was 26.1% in 49 states and the District of Columbia, demonstrating high arthritis prevalence across the US. The median age-standardized prevalence of arthritis was highest in Appalachia. Among adults with arthritis, prevalences of physical inactivity, fair/poor health status, and severe joint pain were highest in the southeastern US. Estimates from BRFSS suggest that while the arthritis prevalence rate has remained stable since 2015 (even as the total number of adults with arthritis increased [7]), physical inactivity among adults with arthritis is declining (8). In 2015, the state median prevalence of adults with arthritis who reported physical inactivity was 35.0% compared with 29.6% in our study; the highest prevalence for both studies was in the southeastern US (8). As of July 2018, the CDC Arthritis Program funds 13 state programs (https://www.cdc.gov/arthritis/partners/funded-states.htm) through a 5-year award to increase awareness of and access to evidence-based interventions for adults living with arthritis, to decrease physical inactivity, and to increase walking. Progress of the funded programs is tracked by monitoring these 3 arthritis-related characteristics, in addition to health care provider physical activity counseling (9), self-management education class attendance (9), and walking for exercise (10).

From 2015 to 2019 the median severe joint pain prevalence reported by adults with arthritis increased slightly, from 29.7% to 32.8% (excluding New Jersey), and is currently highest in the southeastern US (8). Eight states (Alabama, Arkansas, Indiana, Louisiana, Mississippi, South Carolina, Tennessee, and West Virginia) were in the highest quartile for both the prevalence of adults with arthritis reporting severe joint pain and those reporting physical inactivity. The same 8 states were also in the highest quartile for prevalence of fair/poor health status, suggesting a complex interplay between arthritis-attributable joint pain, physical inactivity, and self-rated health status. This interplay represents an opportunity for concentrating efforts to increase promotion and use of evidence-based nonpharmacologic public health interventions in these states.

This study has at least 5 limitations. First, BRFSS data are based on self-report and may be subject to recall, social desirability, and misclassification bias. Social desirability bias may result in prevalence of inactivity being underestimated and recall bias may overestimate or underestimate prevalence of all 3 arthritis-related characteristics, resulting in misclassification of these measures. Second, our findings are not generalizable beyond the noninstitutionalized US adult population aged 18 years or older. Third, low response rates that differ at the state level may bias study findings; however, the weighting methodology attempted to correct this bias. Fourth, the cross-sectional design prevents the assessment of the possible reasons that states have a high or low burden of these arthritis-related measures. Last, the survey question used to identify arthritis prevalence cannot distinguish between numerous arthritis conditions with heterogenous clinical treatments; however, the public health interventions (ie, self-management education and physical activity) are the same for nonpharmacologic treatment of pain.

High burden of physical inactivity, fair/poor health status, and severe joint pain warrant greater awareness and use of arthritis-appropriate, evidence-based public health interventions (https://www.cdc.gov/arthritis/interventions/), such as the Chronic Disease Self-Management Program, Walk with Ease, Fit & Strong, and EnhanceFitness, for adults living with arthritis. The findings presented here highlight the high prevalence of arthritis and the 3 arthritis-related measures, with less favorable health characteristics more prevalent in southeastern states. Further study of differences in sociodemographic and geographic factors of adults with arthritis with high prevalences of these 3 arthritis-related characteristics may help identify groups with a need for arthritis-related interventions.
